# Caesarean Section rate amongst Obstetricians at a tertiary-care hospital of Karachi

**DOI:** 10.12669/pjms.343.14722

**Published:** 2018

**Authors:** Iffat Ahmed, Dure Shahwar, Munazza Akhtar, Azra Amerjee

**Affiliations:** 1 Dr. Iffat Ahmed, FCPS. Department of Obstetrics and Gynaecology, Aga Khan University Hospital, Karachi, Pakistan; 2 Dr. Dur e Shahwar, FCPS. Department of Obstetrics and Gynaecology, Aga Khan University Hospital, Karachi, Pakistan; 3 Dr. Munazza Akhtar, FCPS Department of Obstetrics and Gynaecology, Aga Khan University Hospital, Karachi, Pakistan.; 4 Dr. Azra Amerjee, FCPS, MCPS-HPE, Department of Obstetrics and Gynaecology, Aga Khan University Hospital, Karachi, Pakistan. PGD (Bioethics)

**Keywords:** Caesarean Section, Nullipara, Physician practice, Singleton pregnancy with Vertex presentation, Term

## Abstract

**Objective:**

To analyze the Caesarean Section (CS) rate among different groups of consultants dealing with Nulliparous Term Singleton pregnancy with Vertex (NTSV) presentation delivering at a tertiary care hospital of Karachi over four months.

**Methods:**

This is a secondary analysis of a retrospective data that analyzed factors affecting the CS-rate of NTSV patients. Patients with CS (n=106) were taken as cases and vaginal deliveries (n=106) as controls. This was an unmatched retrospective case-control study.

**Results:**

Mean age of patients was 26.6(SD: 4.2) years. Mean gestational-age was 38.6(SD: 1.0) weeks. Likelihood of CS was slightly less in patients who were attended by feto-maternal consultants(OR:0.81 CI:0.38-1.07) and was slightly more in patients managed by non-full-time faculty (OR:1.04 CI:0.59-1.85). Odds of CS was highest amongst consultants having average monthly volumes of 21-30 patients/month (OR:1.069 CI:0.48-2.34). However none of the above findings were statistically significant. A non-significant increase in risk of CS was observed with increase in experience of physicians (p=0.787).

**Conclusion:**

The results did not show statistically significant difference in CS rate among different groups of Obstetricians. This might indicate that managing labour according to standard guidelines can eliminate physicians’ bias. This can be further evaluated with larger multicenter prospective studies.

## INTRODUCTION

Increasing Caesarean Section (CS) rates are of great concern all over the world.^1^ This imparts increased risk to mothers’ health and life when compared to vaginal deliveries, with no added benefits to fetus. Literature indicates different reasons of rising CS rate. These include increasing rates of labor inductions^2^, inclination of patients for CS, lesser chances of litigation, easy scheduling, and subjectivity of providers’ decision during labour.^3^-^5^ In order to modify the practices and other factors affecting the mode of delivery (MOD)many centers use CS rate as a quality indicator especially among Nulliparous at Term and Singleton pregnancies with Vertex presentation (NTSV).^6^ The rate of CS among NTSV varies widely, from 10.3% to 34.2%.^3^,^7^-^9^ NTSV being low risk is most eligible for vaginal birth. MOD among this group is not only a major determinant of the future obstetrics course^10^, but also reflects the true statistic of CS rate. Indications like major placenta previa, cord prolapse and malpresentation are non-modifiable and are according to standard practices. Variations in practices are seen in conditions like dysfunctional labour and non-reassuring fetal status.^6^ This variation is minimized in developed countries by implementation of guidelines to standardize the clinical practices.^11^

In Pakistan there is no system to gather or monitor national data nor do we have standard guidelines for management of labour. Practices vary among institutes and among consultants. No local data is available but it is observed that very few institutes have set protocols for labour management and even fewer have a system of implementation and audits. The decisions of labour induction and augmentation, use of analgesia during labour, instrumentation and CS are mostly subjective and based on consultants’ discretion. There are few determinants affecting the CS rate like Obstetrician’s experience; volume of patients they were dealing with; working as full-time or non-full-time faculty; and dealing with high or low-risk pregnancies.

The rationale of this analysis was to assess if different characteristics of consultant obstetricians are affecting MOD among NTSV at our hospital. This may help to identify practices among different groups of consultants which can eventually be used to reduce CS rate by modifying these practices. The objective of this study was to analyze the CS rate of different groups of consultants dealing with NTSV women delivering at a tertiary care hospital of Karachi over four months.

## METHODS

This is a secondary analysis of data that has analyzed factors affecting the MOD in NTSV pregnancies.^12^ It was unmatched retrospective case-control study, conducted at a tertiary care hospital in Karachi, Pakistan and had received exemption status from institutional ethical review committee.

NTSV patients admitted between March 1^st^, 2014 and June 30^th^ 2014, for management of labor and delivery were included. Patients delivered through planned caesarean were excluded.

Subjects were identified through computer based medical record system. Data was collected on predesigned proforma (which was validated by three subject specialists). Medical record charts were reviewed. Patients who were delivered by CS were compared with those delivered vaginally (both spontaneous and operative vaginal deliveries). The sample size was calculated with help of WHO sample size calculator and a total of 212 patients were included (106 in each arm), through purposive sampling.

At our center, admitting consultant is the main stakeholder and decision maker in patient care during and after delivery. Labour management is consistent with departmental guidelines. These guidelines are updated according to evidence-based practice, periodically. Continuous Cardiotocography is used to monitor fetal wellbeing and standardized partogram is used to monitor the progress of labour. Details of labour and delivery are entered into a computerized database by the resident who has attended delivery.

Differences in practice were analyzed on basis of following characteristic of consultants:

- ***Experience of consultant in years:*** The experience of a consultant was calculated from the year of passing the post-graduate exam.
- ***Average volume of consultants per month:*** The volume of a consultant was estimated by calculating average number of patients admitted under their care for management of labour and delivery, per month.
- ***Status of consultant (full-time and non-fulltime):*** The appointment status of a consultant as fulltime (FT) and non-fulltime (NFT) differs in terms of the payment schedule and the availability on floor during working hours. FT faculty-members are present on campus during working hours and provide emergency services to all booked/ non-booked patients on rotation. While NFTs work on part-time basis and are accountable for their patients only.
- ***Expertise of consultant:*** High-risk group included feto-maternal consultants; specially trained in fetal and maternal medicine while all other faculty members were included in low-risk group.

Working definitions:

- ***Nullipara:*** Patients who never delivered a baby of >24 weeks or >500gms.
- ***Term pregnancies:*** All pregnancies at or beyond 37 completed weeks are term pregnancies.

### Statistical Analysis

Data was entered, checked and validated, and analyzed by using SPSS 19. The mean of numeric values, including maternal age and gestational age, were calculated. Frequencies of all variables for physician characteristics were reviewed and categorical variables were compared by using chi-square test. Crude Odds ratio and 95% CI was calculated for each characteristic of physician.

## RESULTS

Total of 212 patients were enrolled. This included 106 patients delivered through CS, taken as cases compared with equal number of vaginal deliveries, taken as controls. Patients’ ages ranged from 18 to 39 years, with mean of 26.6 (SD: 4.2) years. Mean gestational age of this study group was 38.6 (SD: 1.0) weeks (Table-I).

**Table-I:** The percentage distribution of variables of NTSV patients (N=212).

Characteristic	N (%)
*Demographic*	
Maternal age (year) Mean ± S.D	26.6 ± 4.2
Gestational Age (Weeks) Mean ± S.D	38.6 ± 1.0
*Physicians dealing with High Risk pregnancies*	
Low Risk	179(84.4)
High Risk	33(15.6)
*Volume of patients (per month)*	
10 or less	23(10.8)
11-20	81(38.2)
21-30	39(18.4)
More than 30	69(32.5)
*Fulltime Faculty*	
Non-fulltime faculty	69(32.5)
Fulltime faculty	143(67.5)
*Experience of Faculty (in years)*	
Up to 12	62(29.2)
13-17	52(24.5)
18-24	68(32.1)
>24	30(14.2)

**Table-II:** Comparison of Caesarean section and Vaginal delivery in NTSV deliveries by physician related parameters.

Characteristic	LSCS n (%)	Vaginal Delivery n (%)	Crude OR (95%CI)	P-value
*Physicians dealing with High Risk pregnancies*				
Low Risk	88 (83)	91 (85.8)	1	0.570
High Risk	18 (17)	15 (14.2)	0.81 (0.38-1.70)	
*Volume of patients (per month)*				
10 or less	09 (8.5)	14 (13.2)	0.589 (0.22-1.54)	0.69
11-20	40 (37.7)	41 (38.7)	0.894 (0.47-1.70)	
21-30	21 (19.8)	18 (17)	1.069 (0.48-2.34)	
More than 30	36 (34)	33 (31.1)	1	
*Fulltime Faculty*				
Fulltime faculty	71 (67)	72 (67)	1	0.883
Non-fulltime faculty	35 (33)	34 (32)	1.04 (0.59-1.85)	
*Experience of Faculty (in years)*				
Up to 12	31 (29.2)	31 (29.2)	1	0.787
13-17	23 (21.7)	29 (27.4)	0.79 (0.38- 1.67)	
18-24	36 (34)	32 (30.2)	1.12 (0.56- 2.24)	
>24	16 (15.1)	14 (13.2)	1.14 (0.48- 2.73)	

As seen in Table-I, only16% patients were delivered under care of consultants dealing with high-risk pregnancies. Maximum number of patients (n=81, 38.2%) were delivered by consultants with volumes of 11-20 deliveries per month (quartile 2). FT faculty dealt with 67.5% of cases while 32.5% were delivered by NFT consultants. Doctors with 18-24 years’ experience (quartile 3) dealt with 68(32.1%) patients. The likelihood of CS in an NTSV patient attended by a feto-maternal consultant was slightly less (OR 0.81 CI: 0.38-1.07) although this was not statistically significant (p-value: 0.570).

When obstetricians were compared on the basis of their average monthly volumes of patients, the Odds of CS, as compared to vaginal delivery, was highest among those consultants having 21-30 deliveries per month (OR 1.069 CI: 0.48-2.34) than those having >30 deliveries per month on an average. However, these associations were not significant (p-value 0.696).

The CS rate in patients managed by NFT was slightly higher when compared with those delivered vaginally (OR1.04 CI: 0.59-1.85). This was however not statistically significant (p value: 0.883).

The Odds of CS increased slightly with increase in experience of physicians. Although statistically, this was not significant (p-value: 0.787). Univariate correlation did not show any differences in practices of different groups therefore we are not reporting multivariate analysis.

## DISCUSSION

Our study showed similar results of CS rate among different groups of consultants when dealing with NTSV patients. This was a retrospective study and it was assumed that the available data was correct. Moreover the confounding factor of different teams involved in the management of cases was overlooked, assuming that the input of instructors’ and residents’ equally affected the results of all consultants.

The results of our study were similar to Manohar S et al., and showed that rates of normal and interventional deliveries were not different among different consultants.^11^ That study compared vaginal delivery with interventional (instrumental and caesarean) deliveries while our study grouped normal and instrumental deliveries as vaginal delivery to compare it with abdominal delivery.

As the feto-maternal specialists deal with high risk patients having medical or surgical co-morbids, the risk of CS is expected to be higher in this group as compared to other consultants.^6^ The CS rate, in our study was slightly lesser among high-risk group of consultants though this was not statistically significant. This may be because, we selected term pregnancies and several high risk patients may need delivery before 37 completed weeks.

Consultants with higher volumes may be more comfortable in managing normal labour and have been reported to have lesser number of CS as compared to obstetrician with low volumes.^6^,^13^ In our study CS rates among all volume quartiles were almost constant. As also seen by Clapp MA et al. the experience of obstetricians had no effect on the MOD in our patients.^13^

There is a general impression in lay public that tendency of labour-induction, expedition of deliveries and low threshold for CS is higher when labour is managed by NFT consultants. This opinion is based on the premise that these obstetricians deal with laboring patients at multiple places simultaneously and therefore spend lesser time at one centre.^6^ This study showed a non-significant increase in CS rate in patients of NFT faculty and was concordant with the finding of McClelland S et al.^6^

The practices of different obstetrician groups and possibility of CS among their patients were found to be similar in this study. This may be because labour is managed according to standard guidelines in our institute with regular audits and monitoring of dash-board indicators.^11^ We therefore recommend future prospective studies to identify the causes of increased caesarean section rates in our institute. However this study may contribute to local data regarding different practices among obstetricians, but being a secondary analysis of a retrospective data from a single institute it has its limitation.

## CONCLUSION

The results did not show statistically significant difference in CS rate among different groups of Obstetricians. This might indicate that managing labour according to standard guidelines can eliminate physicians’ bias. This can be further evaluated with larger multicenter prospective studies.

## References

[1] Kozhimannil KB, Law MR, Virnig BA (2013). Cesarean delivery rates vary tenfold among US hospitals;reducing variation may address quality and cost issues. Health Aff (Millwood).

[2] Luthy DA, Malmgren JA, Zingheim RW (2004). Cesarean delivery after elective induction in nulliparous women:the physician effect. Am J Obstet Gynecol.

[3] Kottmel A, Hoesli I, Traub R, Urech C, Huang D, Leeners B (2012). Maternal request:a reason for rising rates of cesarean section?. Arch Gynecol Obstet.

[4] Kingdon C, Baker L, Lavender T (2006). Systematic review of nulliparous women's views of planned cesarean birth:the missing component in the debate about a term cephalic trial. Birth.

[5] King JF (2000). Obstetric interventions among private and public patients. High rates of operative vaginal interventions in private patients need analysis. BMJ.

[6] McClelland S, Gorfinkle N, Arslan AA, Benedetto-Anzai MT, Cheon T, Anzai Y (2017). Factors associated with cesarean delivery rates:a single-institution experience. Matern Health Neonatol Perinatol.

[7] Sharma V, Colleran G, Dineen B, Hession MB, Avalos G, Morrison JJ (2009). Factors influencing delivery mode for nulliparous women with a singleton pregnancy and cephalic presentation during a 17-year period. Eur J Obstet Gynecol Reprod Biol.

[8] Liu S, Rusen ID, Joseph KS, Liston R, Kramer MS, Wen SW (2004). Recent trends in caesarean delivery rates and indications for caesarean delivery in Canada. J Obstet Gynaecol Can.

[9] Joseph KS, Young DC, Dodds L, O'Connell CM, Allen VM, Chandra S (2003). Changes in maternal characteristics and obstetric practice and recent increases in primary cesarean delivery. Obstet Gynecol.

[10] Litorp H, Kidanto HL, Nystrom L, Darj E, Essen B (2013). Increasing caesarean section rates among low-risk groups:a panel study classifying deliveries according to Robson at a university hospital in Tanzania. BMC Pregnancy Childbirth.

[11] Manohar S, Woods CF, Lindow SW (2015). Individual consultant practice does not affect the overall intervention rate:a 6-year study. J Perinat Med.

[12] Ahmed I, Chishti U, Akhtar M, Ismail H (2016). Factors affecting mode of delivery in a nullipara at term with singleton pregnancy and vertex presentation (NTSV). Pak J Med Sci.

[13] Clapp MA, Melamed A, Robinson JN, Shah N, Little SE (2014). Obstetrician volume as a potentially modifiable risk factor for cesarean delivery. Obstet Gynecol.

